# GPi DBS treatment outcome in children with monogenic dystonia: a case series and review of the literature

**DOI:** 10.3389/fneur.2023.1151900

**Published:** 2023-04-24

**Authors:** Darko Chudy, Marina Raguž, Vladimira Vuletić, Valentino Rački, Eliša Papić, Nataša Nenadić Baranašić, Nina Barišić

**Affiliations:** ^1^Department of Neurosurgery, Dubrava University Hospital, Zagreb, Croatia; ^2^Department of Surgery, School of Medicine, University of Zagreb, Zagreb, Croatia; ^3^School of Medicine, Catholic University of Croatia, Zagreb, Croatia; ^4^Department of Neurology, School of Medicine, University of Rijeka, Rijeka, Croatia; ^5^Department of Pediatrics, University Hospital Centre, School of Medicine, University of Zagreb, Zagreb, Croatia

**Keywords:** pediatric genetic dystonia, DYT6 gene, *KMT2B* dystonia, GPi-DBS, neurosurgery

## Abstract

**Introduction:**

Dystonia is the third most common pediatric movement disorder and is often difficult to treat. Deep brain stimulation (DBS) of the internal pallidum (GPi) has been demonstrated as a safe and effective treatment for genetic dystonia in adolescents and adults. The results of DBS in children are limited to individual cases or case series, although it has been proven to be an effective procedure in carefully selected pediatric cohorts. The aim of our study was to present the treatment outcome for 7- to 9-year-old pediatric patients with disabling monogenic isolated generalized DYT-*THAP1* and DYT-*KMT2B* dystonia after bilateral GPi-DBS.

**Patients and results:**

We present three boys aged <10 years; two siblings with disabling generalized DYT-*THAP1* dystonia and a boy with monogenic-complex DYT-*KMT2B*. Dystonia onset occurred between the ages of 3 and 6. Significantly disabled children were mostly dependent on their parents. Pharmacotherapy was inefficient and patients underwent bilateral GPi-DBS. Clinical signs of dystonia improved significantly in the first month after the implantation and continued to maintain improved motor functions, which were found to have improved further at follow-up. These patients were ambulant without support and included in everyday activities. All patients had significantly lower Burke–Fahn–Marsden Dystonia Rating Scale (BFMDRS) values, indicating >25% improvement over the first 15 months. However, there was a decline in speech and upper limb function, manifesting with bradylalia, bradykinesia, and dysphonia, which decreased after treatment with trihexyphenidyl.

**Conclusion:**

Although reports of patients with monogenic dystonia, particularly DYT-*THAP1*, treated with DBS are still scarce, DBS should be considered as an efficient treatment approach in children with pharmacoresistent dystonia, especially with generalized monogenic dystonia and to prevent severe and disabling symptoms that reduce the quality of life, including emotional and social aspects. Patients require an individual approach and parents should be properly informed about expectations and possible outcomes, including relapses and impairments, in addition to DBS responsiveness and related improvements. Furthermore, early genetic diagnosis and the provision of appropriate treatments, including DBS, are mandatory for preventing severe neurologic impairments.

## Introduction

Dystonia (DYT) is “*a movement disorder characterized by sustained involuntary or intermittent muscle contractions causing abnormal, sustained, often repetitive movements, postures, or both”* (1). Dystonia may be focal, segmental, or generalized, resulting in twisting, sustained, and repetitive postures and movements, with the progressive development of severe motor disability and a negative impact on quality of life (1). The etiology, pathophysiology, and clinical presentation are heterogeneous, ranging from pediatric-onset to adult-onset generalized dystonia, with the possible development of life-threatening dystonic storm. In the last couple of decades, a great number of genes have been identified and linked to different types of dystonia, such as torsin family 1 member A (*TOR1A*) in 1997 [linked to dystonia type 1 [DYT-[*TOR1A*]] and thanatos-associated-domain containing apoptosis-associated protein 1 (*THAP1*) in 2009 [linked to dystonia type 6 with onset occurring usually during childhood or adolescence, including segmental or generalized dystonia with initial craniocervical or laryngeal and upper limb involvement (2–4)]. In addition, since 2016, several mutations in the lysine methyltransferase 2B (*KMT2B*) gene have been identified and linked to progressive childhood-onset dystonia, including development from focal toward generalized dystonia with pronounced craniofacial and laryngeal involvement (5).

Deep brain stimulation (DBS) of the internal globus pallidum (GPi) has been demonstrated as a safe and effective treatment for primary dystonia with a genetic cause, such as DYT-*TOR1A* and DYT-*THAP1* in adolescents and adults (6, 7). The number of adolescents and adults with DYT-*THAP1* dystonia who underwent GPi DBS to date is relatively small. The first published small case series, including several patients, reported only moderate responses to DBS (8, 9). However, medically refractory DYT-*THAP1* cases receiving GPi DBS showed favorable outcomes, more similar to those observed in other isolated dystonias (9–16).

Dystonia is the third most common pediatric movement disorder and is often difficult to treat (17). Coubes et al. were the first to report on the treatment of a child with dystonia using bilateral GPi-DBS (18). In the last decade, a number of reports of DBS in children have been published, showing excellent outcomes for monogenic isolated dystonia DYT-*TOR1A*, as well as the general consensus that DBS is safe and effective in the treatment of adults (7, 19–21). The results of DBS in children are limited to individual cases or case series, although DBS for childhood dystonia has been proven to be an effective procedure in carefully selected pediatric cohorts. Although it is suggested that DBS should be considered as a treatment approach in pharmacoresistent DYT-*THAP1* early in childhood to prevent more severe symptoms, such as disabling motor development and quality of life decline, including emotional and social aspects, the results are missing. Additionally, bilateral GPi-DBS has been reported as a valuable therapeutic option for DYT-*KMT2B*, especially for regaining motor function and mobility, but less so for speech if significant speech difficulties are developed (5, 22–37).

Thus, the aim of our study was to present efficient treatment outcomes of two siblings with DYT-*THAP1* and a boy with DYT-*KMT2B* with disabling monogenic generalized dystonia after bilateral GPi DBS in childhood.

## Patients and results

We present three boys: two siblings at the of 9 (patient I) and 7 (patient II) years of age with DYT-*THAP1* and a boy of 7 years of age (patient III) with DYT-*KMT2B* who had DBS implanted.

### Case 1

A boy was born after an uneventful pregnancy and birth; both the mother and father were non-consanguineous, with a negative family history for neurologic disorders. His development was normal until the age of three; he became clumsy, with coordination disturbances and lordotic posturing, with hypotonia, torticollis to the right side, and slurred speech. The initial extensive hospital diagnostic investigations excluded structural, infectious, autoimmune, metabolic, chromosomal, and paraneoplastic pathological conditions; however, the diagnosis and etiology remained unknown. No psychiatric or cognitive dysfunctions or disturbances were associated. Over 7 years, the progressive impairment of clinical signs occurred and he developed generalized torsion-type dystonia and became wheelchair-bound. His speech was severely affected as evidenced by slurring, and exhibited upper limb dysfunction with associated dyskinetic movements and tremor. On exam at admission, the boy was not ambulant and had generalized dystonia at rest and during activity, as well as dysphonic and dysarthric speech along with swallowing difficulties (Supplementary Video 1). A dystonia gene panel using PCR amplification and sequence analysis revealed a *THAP 1*, C270_273del (p.glu91ilefs^*^28) mutation. The patient was treated with clonazepam, trihexyphenidyl, and baclofen, but without meaningful improvement. The Burke–Fahn–Marsden Dystonia Rating Scale-movement scale (BFMDRS-M) was 116 and the BFMDRS-disability scale (BFMDRS-D) was 28 (Table 1).

**Table 1:** Preoperative characteristics of dystonia patients included in the study.

**No**.	**Gender**	**Age at disease onset (years)**	**Initial anatomical distribution**	**Sequence variant**	**Preop BFMDRS-M**	**Preop BFMDRS-D**	**Preop medication**
I	M	3	Upper limb dystonia and slurred speech	THAP 1, C270_273del (p.glu91ilefs*28)	115	28	Clonazepam, tryhexyphenidyl, and baclofen
II	M	6	Upper limb dystonia and slurred speech	THAP 1, C270_273del (p.glu91ilefs*28)	96	20	Clonazepam, tryhexyphenidyl, and baclofen
III	M	4.5	Speech developmental delay, anarthria, and lower limb dystonia	KMT2B c.5572dupC; pArg1858Profs * 114	106	22	Valproat, clobazam, ethosuximide, levetiracetam, and petinimid

### Case 2

Patient 2, a male sibling of patient I, born after normal pregnancy and delivery. His motor, mental, speech, and cognitive development were normal. He started to walk unassisted at the age of 12 months. At the age of 4.5 years, he manifested with involuntary dystonic movements of the first upper limbs, and afterwards he became clumsy and could not run without frequent falls. On exam, bradylalia and dysarthria were present, with oromandibular dystonia, resulting in scarce verbal response. He could not maintain left side upper and lower extremities in antigravity positions nor walk downstairs without assistance. He also experienced gear phenomenon on lower limbs and waddling gait, absent tendon reflexes of the left side. He gradually developed generalized dystonia manifested at rest and provoked by activity (Supplementary Video 2). No psychiatric or cognitive dysfunction were registered other than slurred speech. A dystonia gene panel revealed a *THAP1*, C270_273del (p.glu91ilefs^*^28) mutation, the same as in his brother (patient 1). Treatment with trihexyphenidyl, clonazepam, and baclofen was introduced but without clinical significance. The preoperative BFMDRS-M was 96 and the BFMDRS-D was 20 (Table 1).

### Case 3

Patient 3, a boy was born after an uneventful pregnancy, with the umbilical cord wrapped around his neck during childbirth. The Apgar score was 9/10 and he was discharged from hospital as a healthy newborn. He started to walk unassisted at the age of 12 months. Pronounced developmental speech delay was significant. At the age of 3, his speech was totally undeveloped and he manifested with anarthria. Motor development except speech was normal until the age of 3.5 years when tremor, chorea, and walking difficulties occurred; his gait became clumsy and ataxic, and he often fell. He could run only backwards with trunk rotation, and manifested occasional jerky arm tremor associated with attention deficit disorder. On exam, thoracic kyphosis, choreoathetoid movements, occasional jerky hand tremor, and coordination disturbances were observed. The patient could not hold extremities in antigravity positions due to muscle weakness. At the time he also manifested with significant speech developmental delay and anarthria. Owing to delayed speech development, EEG was recorded and revealed bilateral multifocal epileptiform discharges. Treatment with antiepileptics, including valproate, clobazam, ethosuximide, levetiracetam and petinimid, was introduced. A brain MRI showed small hypothalamic hamartoma and lipoma, and a conservative approach was suggested. The patient manifested further progressive speech regression, upper limb tremor, bizarre walking patterns, severe hand tremor, and anarthria (Supplementary Video 3). Diagnostic work up except brain MR and EEG was normal (EMNG, VEP and BAER, cerebrospinal fluid, and metabolic analysis), including spinal MRI and brain SPECT. A gene panel for dystonia revealed a pathogenic *de novo* variant in gene *KMT2B* c5572dupC;p.Arg1858Profs^*^114. The BFMDRS-M was in total 106 and the BFMDRS-D was 22 (Table 1).

All patients underwent bilateral GPi-DBS at the age 7 and 9. After surgical planning using anatomical targeting through preoperative frameless MRI and CT obtained with a mounted Leksell frame (Electa, Stockholm, Sweden) on patients head the electrodes have been implanted bilaterally in GPi (11). In patient I, Activa RC neurostimulator and model 3389 electrodes (Medtronic) were implanted, whereas in patients II and III, the Vercise rechargeable neurostimulator and directional electrodes (Boston Scientific) were implanted. The stimulation started with the following parameters: for patient I an amplitude of 1.9 V, a frequency of 125 Hz, and a pulse duration of 60 μs, and for patient II and III, an amplitude of 2 mA, a frequency of 130 Hz, and a pulse duration of 60 μs. The pulse amplitude was gradually increased over the 6 months following implantation.

On the first follow up, the first patient's signs improved significantly, enabling the patient to sit independently and to walk with the assistance of one person (Supplementary Video 1). In the weeks and months following the DBS procedure, he regained some manual abilities (he became able to write with moderate difficulty), discrete speech improvement was registered (dysarthria and dysphonia were still present and now bradylalia), mobility (he walked with minimal assistance), and could perform basic self-care tasks with some help (feeding, dressing, etc.). In general, his quality of life improved dramatically. The BFMDRS-M decreased to 64 after GPi DBS, and this persisted after more than 2 years of follow up, while the BFMDRS-D decreased to 8 and persisted (Table 2). However, dysphonia, bradykinesia, bradylalia, fatiguability, and dystonic posturing were still present and mildly increased 2 years after implantation.

**Table 2:** Outcome after GPi DBS of dystonia patients included in the study.

**No**.	**Age at GPi DBS (years)**	**Disease duration prior to GPi DBS (years)**	**Follow up (years, months)**	**Change in BFMDRS-M, last follow-up compared to baseline**	**Change in BFMDRS-D, last follow-up compared to baseline**	**Responder (>25% improvement)**	**Effect on speech and/or swallowing**	**Long-term follow-up**	**DBS parameters at last follow up**
I	9	6	2 years, 8 months	115 > 64	28 > 8	✓	✓	Bradykinesia and bradylalia (after 24 months)	•GPi L (8+, 9–, 10+) 3.9 mA, 60 μs, 180 Hz •GPi R (0+, 1–, 2+) 4.9 mA, 60 μs, 180 Hz
II	7	1	1 year	96 -> 50	20 > 9	✓	✓	No impairment	•GPi L (C+, L3-10%, L4-11%, L5-18%, L6-53%, L7-8%) 2.8 mA, 90 μs, 113 Hz •GPi R (C+, L1-1%, L4-22%, L5-62%, L6-6%, L7-9%) 3.6 mA, 90 μs, 113 Hz
III	7	2	1 year 11 months	106 -> 67	22 > 12	✓	+/-	Dysphonia (dys/anarthria), swallowing and chewing difficulties, and freezing of the gait impairment (after 15 months)	•GPI L (C+, L1-20%, L2 2–22% 3–38%, L3 5–20%) 4.5 mA, 100 μs, 130 Hz •GPI R (C+, L1-11%, L2 2–22% L3 5–13%, 6–28%) 4.0 mA, 90 μs, 130 Hz

Twelve months after DBS implantation, patient II had only mild and occasional upper limb dystonia, a stable gait, was able to run fast, had a stronger voice, and had more fluent and understandable speech (Supplementary Video 2). The BFMDRS-M decreased to 50 after GPi DBS, and this persisted after almost 1 year of follow up, while the BFMDRS-D decreased to 9 and persisted (Table 2).

After DBS implantation, only transitory but very discrete speech improvement occurred in patient III (although it was agrammatic and dysphonic), whose upper limb function and gait improved so he was able to walk unaided (Supplementary Video 3). His dystonia was worsened by specific activities (such as running, walking, chewing, swallowing, writing, and less targeting the objects) and he still had some cognitive and behavioral dysfunctions issues. His follow up EEG showed pronounced and intensive bilateral focal and multifocal discharges. His AEDs were tapered off prior to DBS implantation. He manifested freezing of the gait 15 months after DBS and developed anarthria, and again manifested with upper limb dystonia during activity associated with swallowing and bolus clearance difficulties. The BFMDRS-M scale decreased to 58 after GPi DBS. After 6 months, as the progression of dystonia occurred, the BFMDRS-M score increased to 67 but still showed significant improvement, while the BFMDRS-D decreased to 12 (Table 2). During postoperative follow up no complications occurred in any of the presented patients.

## Literature review

We did not follow the exact methodology of a systematic review, i.e., we performed a PubMed Search using the terms “*KMT2B*” or “*THAP1”* and “*dystonia*” and “*Deep Brain stimulation”*.

In the period between 2010 and 2022, we identified 10 studies, including 38 patients with DYT-*THAP1* who underwent GPi DBS describing significant improvements in both BFMDRS-M and BFMDRS-D (Supplementary Table 1). Furthermore, in the period between 2017 and 2022, we identified 16 studies, including 64 patients with DYT-*KMT2B* who underwent DBS (Supplementary Table 2). In the vast majority of cases, the lead was implanted in the GPi bilaterally, and STN-DBS was only performed in one patient. Furthermore, during follow-up, significant changes were described in BFMDRS-M and BFMDRS-D, ranging from 20% to more than 95% improvement.

## Discussion

We present two siblings with DYT-*THAP1* and a child with DYT-*KMT2B*, all pharmacoresistent with remarkable clinical improvement after GPi-DBS.

DYT-*THAP1* is an early-onset initially craniofacial and later typical generalized dystonia with autosomal-dominant inheritance, rostrocaudal progression, and a sex-independent penetrance of 60%, with causative mutations in the *THAP1* gene on chromosome 8 (38). The *THAP1* gene is part of a family of THAP proteins that bind specific DNA sequences and regulate cell proliferation through pRB/E2F cell cycle target genes, a pathway recently proposed to be involved in cell death in Parkinson's disease (21, 39). The phenotype of patients with DYT-*THAP1* is highly variable, with age of onset ranging from 8 to 69 years and the site of onset predominantly cervical, laryngeal, and in the upper limbs, and associated with tremor and signs of parkinsonism in some patients. The involvement of cranial muscles, leading to disabling dysarthria or dysphonia, as was also the case in our patients, is typical for DYT-*THAP1* (40).

DYT-*KMT2B* is a progressive childhood-onset disorder, evolving from a focal to a generalized pattern, either from lower-limb dystonia in the first decade of life, which accounts for up to 10% of cases, or first affecting the laryngopharyngeal region, speech development, and upper limb function, and developing into generalized dystonia in a craniocaudal manner (41). Gene mutations occur *de novo*, and are usually non-sense mutations, rather than missense mutations. DYT-*KMT2B* is often associated with endocrinological symptoms, short stature, early onset with a median of 5 years of age, laryngeal dystonia, and onset on lower extremities. Developmental and pronounced delay in speech development, as in our patients, precede dystonia in 30% of patients (25). Further clinical characteristics, such as cognitive disability, psychiatric comorbidities, and dysmorphic features, have been reported in several patients (27–29). Bilateral GPi-DBS has been reported as an efficient therapeutic option, especially for improving movement disorder and regaining independent mobility (5, 22–37). GPi-DBS is sometimes associated with “dramatic” amelioration in gait but more commonly associated with truncal dystonia and scoliosis; it is rarely associated with speech dysfunction in severely affected patients with KMT2B gene mutations prior to DBS (23).

The first-line treatment for multifocal or generalized dystonia is trihexyphenidyl and baclofen, which provide limited and transient improvement in some patients. Intramuscular injections of botulinum toxin A are used mainly for the treatment of focal dystonia (42). L-dopa at low doses (50–200 mg) represents the first-line treatment for dopa-responsive dystonia. AnT empirical trial of L-dopa is usually offered to all patients with early-onset dystonia without evidence of neurodegeneration or brain structural lesions, partly due to a delay in treatment onset and a lack of feasibility of next-generation sequencing and the genetic screening of dystonia (42).

As already mentioned, the results of DBS in children are limited to individual cases or case series, although DBS for childhood dystonia has been proven to be an effective procedure in carefully selected pediatric cohorts. A positive response to treatment has been demonstrated in DYT-*TOR1A* patients after GPi-DBS (43, 44). Several adult and pediatric patients underwent GPi-DBS due to DYT-*THAP1* and DYT-*KMT2B*. Although the number of pediatric patients was lower, the results prove that bilateral GPi-DBS is a valuable therapeutic option for DYT-*THAP1* (8–16) and DYT-*KMT2B*, particularly for movement disorders and regaining mobility, but less so for speech issues (5, 22–37) (Supplementary Tables 1, 2). So far, the aforementioned patients presented with severe dystonic postures, majorly impacting their quality of life, as well as those of their families and caregivers. In the vast majority of patients who underwent bilateral GPi-DBS, a significant improvement was reported in both BFMDRS-M and BFMDRS-D, ranging from 20% to more than 95% during follow-up, from which the longest was 22 years (Supplementary Tables 1, 2). Systematic review and meta-analysis of GPi-DBS for DYT-*KMT2B* with a median follow-up of 12 months reported a 42% improvement in the BFMDRS scale, with better outcomes with more severe dystonia at baseline (24), which is compatible with our results.

Although highly effective in some patients, it is known that the stimulation response can be variable and difficult to predict, emphasizing the need for controlled studies in pediatric cohorts exclusively (7, 19, 20). As presented in the literature, stimulation parameters vary widely among patients; therefore, optimal programming is individual (Supplementary Tables 1, 2).

Furthermore, genetic or acquired causes of dystonia and other movement disorders lead to the development of basal ganglia, thalamus, cortex, dentate cerebellar nucleus, and brain stem lesions or dysfunction (45). GPi-DBS can affect neuronal activity in functional connections of the cortico-basal ganglia neural network and cause long-term plasticity changes at the cortical level, which can then reestablish normal movement (46, 47). Additionally, beside modulation in neuronal networks, DBS may play an important role in the neurochemical system, e.g., modulating neurotransmitter (dopamine, glutamate, and gamma-aminobutyric acid) release (46, 48). It is known that various genetic etiologies and dystonia pattern respond differently to DBS (49). The best results are achieved in DYT-*THOR1*. DBS efficiency in DYT-*THAP1* has been described as variable but improvement occurs in 50–70% of patients. Early identification of a genetic etiology for dystonia is critical because a correct diagnosis can ensure timely and appropriate treatment, such as DBS, before disability or deformity occurs (6). Impairments after initial improvement could be the result of either associated or induced parkinsonism related to specific gene mutation or induced after DBS implantation (50). Mild parkinsonian signs are an additional manifestation of dystonia arising from basal ganglia dysfunction. In DYT-*KMT2B* patients, decline is progressive after 7–22 years of post DBS implantation assessment (31).

Several studies have investigated the potential predictive factors (i.e., biomarkers) of DBS treatment outcome for early-onset dystonia in pediatric population. It was shown that the age at onset of dystonia, severity, and previous duration of disease and not age at surgery are associated with good treatment response and outcome, while a shorter time between diagnosis and DBS was significant. Although some studies did not show a correlation of preoperative dystonia severity and DBS efficacy, others indicated that a lower preoperative BFMDRS score is related to better treatment efficacy and outcome (20, 47). In addition, DBS treatment outcome is less efficient if speech has been severely affected prior to DBS implantation, if the disease duration is longer, and if disease onset is later; the outcome is also less efficient for complex or combined monogenic dystonia (20, 21). Factors such as age at onset, disease duration, specific gene mutation, dystonia pattern, and severity of preoperative dystonia will definitely be included and discussed in future studies. A better understanding of outcomes in pediatric dystonia is leading to the refinement of the indications for DBS, which continue to evolve (20, 21). Nevertheless, GPi-DBS should be considered in a timely manner once the symptoms cannot be controlled by medications.

## Conclusions

Although the number of patients with dystonia, particularly DYT-*THAP1*, treated with GPi-DBS is still small, we believe that DBS should be considered in patients as an early treatment approach in pediatric pharmacoresistent dystonia to prevent more severe symptoms decreasing the quality of life, including emotional and social aspects. Parents should be properly informed about the expected and possibly variable improvement after DBS implantation. Using whole-exome sequencing, it is possible to establish the correct diagnosis early, which can be helpful in determining the appropriate treatment, such as DBS, before disability or deformity occur. Furthermore, we emphasize the importance of genetic analysis in pediatric dystonia patients, particularly those with monogenic isolated generalized dystonia with normal brain MR, who are possible candidates for DBS treatment, especially because the identification of an underlying molecular defect could significantly help predict the efficacy and functional outcome of DBS. DBS treatment outcome is less efficient if speech has been severely affected prior to DBS implantation and in complex or combined monogenic dystonia.

## Data availability statement

The original contributions presented in the study are included in the article/Supplementary material, further inquiries can be directed to the corresponding author.

## Ethics statement

Written informed consent was obtained from the individual(s) and/or minor(s)' legal guardian/next of kin for the publication of any potentially identifiable images or data included in this article.

## Author contributions

DC, NB, and VV contributed equally to the conception and design of the study, investigation, and formal data analysis. NB, MR, VR, and EP organized the data and prepared Supplementary material. NB and DC wrote the first draft of the manuscript. VV, NN, NB, and MR wrote sections of the manuscript. All authors contributed to manuscript revision and read and approved the submitted version.

## References

[1] Albanese A Bhatia K Bressman SB Delong MR Fahn S Fung VS. Phenomenology and classification of dystonia: a consensus update. Mov Disord. (2013) 28:863–73. 10.1002/mds.25475 23649720 PMC3729880

[2] Fuchs T Gavarini S Saunders-Pullman R Raymond D Ehrlich M Bressman S. Mutations in the THAP1 gene are responsible for DYT6 primary torsion dystonia. Nat Genet. (2009) 41:286–8. 10.1038/ng.304 19182804

[3] Xiromerisiou G Houlden H Scarmeas N Stamelou M Kara E Hardy J. THAP1 mutations and dystonia phenotypes: genotype phenotype correlations. Mov Disord. (2012) 27:1290–4. 10.1002/mds.25146 22903657 PMC3664430

[4] Djarmati A Schneider SA Lohmann K Winkler S Pawlack H Hagenah J. Mutations in THAP1 (DYT6) and generalized dystonia with prominent spasmodic dysphonia: a genetic screening study. Lancet Neurol. (2009) 8:447–52. 10.1016/S1474-4422(09)70083-3 19345148

[5] Zech M Lam DD Winkelmann J. Update on KMT2B-Related Dystonia. Curr Neurol Neurosci Rep. (2019) 19:92. 10.1007/s11910-019-1007-y 31768667

[6] Holloway KL Baron MS Brown R Cifu DX Carne W Ramakrishnan V. Deep brain for dystonia: a meta-analysis. Neuromodulation. (2006) 9:253–61. 10.1111/j.1525-1403.2006.00067.x 22151759

[7] Vidailhet M Vercueil L Houeto JL Krystkowiak P Lagrange C Yelnik J. Bilateral, pallidal, deep-brain stimulation in primary generalised dystonia: a prospective 3 year follow-up study. Lancet Neurol. (2007) 6:223–9. 10.1016/S1474-4422(07)70035-2 17303528

[8] Groen JL Ritz K Contarino MF van de Warrenburg BP Aramideh M Foncke EM. DYT6 dystonia: mutation screening, phenotype, and response to deep brain stimulation. Mov Disord. (2010) 25:2420–7. 10.1002/mds.23285 20687191

[9] Panov F Tagliati M Ozelius LJ Fuchs T Gologorsky Y Cheung T. Pallidal deep brain stimulation for DYT6 dystonia. J Neurol Neurosurg Psychiatry. (2012) 83:182–7. 10.1136/jnnp-2011-300979 21949105

[10] Jech R Bareš M Krepelová A Urgošík D Havránková P RuŽička E. 6-a novel THAP1 mutation with excellent effect on pallidal DBS. Mov Disord. (2011) 26:924–5. 10.1002/mds.23599 21425341

[11] Krause P Brüggemann N Völzmann S Horn A Kupsch A Schneider GH. Long-term effect on dystonia after pallidal deep brain stimulation (DBS) in three members of a family with a THAP1 mutation. J Neurol. (2015) 262:2739–44. 10.1007/s00415-015-7908-z 26486352

[12] Vuletic V Chudy D Almahariq F Dobricic V Kostic V Bogdanovic N. Excellent outcome of pallidal deep brain stimulation in DYT6 dystonia: a case report. J Neurol Sci. (2016) 366:18–9. 10.1016/j.jns.2016.04.032 27288769

[13] Danielsson A Carecchio M Cif L Koy A Lin JP Solders G. Pallidal deep brain stimulation in DYT6 dystonia: clinical outcome and predictive factors for motor improvement. J Clin Med. (2019) 8:2163. 10.3390/jcm8122163 31817799 PMC6947218

[14] Tai CH Lee WT Tseng SH. DYT6 dystonia mimicking adolescent idiopathic scoliosis successfully treated by pallidal stimulation. Int Med Case Rep J. (2021) 14:315–21. 10.2147/IMCRJ.S307010 34012300 PMC8128503

[15] Sankhla CS Sankhe M Ray J. Long-term efficacy of pallidal deep brain stimulation in a patient with DYT-THAP1 (DYT-6) dystonia from India. Ann Indian Acad Neurol. (2022) 25:314–6. 10.4103/aian.aian_378_21 35693669 PMC9175436

[16] Grofik M Cibulka M Olekšáková J Turčanová Koprušáková M Galanda T. A case of novel DYT6 dystonia variant with serious complications after deep brain stimulation therapy: a case report. BMC Neurol. (2022) 22:344. 10.1186/s12883-022-02871-3 36096774 PMC9465909

[17] Cif L Coubes P. Historical developments in children's deep brain stimulation. Eur J Paediatr Neurol. (2017) 21:109–17. 10.1016/j.ejpn.2016.08.010 27693334

[18] Coubes P Echenne B Roubertie A Vayssiere N Tuffery S. Traitement de la dystonie généralisée à début précoce par stimulation chronique bilatérale des globus pallidus internes. A propos d'un cas. Neurochirurgie. (1999) 45:139–44. 10448655

[19] Air EL Ostrem JL Sanger TD Starr PA. Deep brain stimulation in children: experience and technical pearls. J Neurosurg Pediatr. (2011) 8:566–74. 10.3171/2011.8.PEDS11153 22132914

[20] Elkaim LM Alotaibi NM Sigal A Alotaibi HM Lipsman N Kalia SK. Deep brain stimulation for pediatric dystonia: a meta-analysis with individual participant data. Dev Med Child Neurol. (2019) 61:49–56. 10.1111/dmcn.14063 30320439

[21] Tisch S Kumar KR. Pallidal deep brain stimulation for monogenic dystonia: the effect of gene on outcome. Front Neurol. (2021) 11:630391. 10.3389/fneur.2020.630391 33488508 PMC7820073

[22] Mun JK Kim AR Ahn JH Kim M Cho JW Lee JI. Successful pallidal stimulation in a patient with KMT2B-related dystonia. J Mov Disord. (2020) 13:154–8. 10.14802/jmd.19087 32241076 PMC7280936

[23] Abel M Pfister R Hussein I Alsalloum F Onyinzo C Kappl S. Deep brain stimulation in KMT2B-related dystonia: case report and review of the literature with special emphasis on dysarthria and speech. Front Neurol. (2021) 12:662910. 10.3389/fneur.2021.662910 34054706 PMC8160374

[24] Rajan R Garg K Saini A Radhakrishnan DM Carecchio M Bk B. GPi-DBS for KMT2B-associated dystonia: systematic review and meta-analysis. Mov Disord Clin Pract. (2022) 9:31–7. 10.1002/mdc3.13374 35005062 PMC8721835

[25] Meyer E Carss KJ Rankin J Nichols JM Grozeva D Joseph AP. Mutations in the histone methyltransferase gene KMT2B cause complex early-onset dystonia. Nat Genet. (2017) 49:223–37. 10.1038/ng.3740 27992417

[26] Kawarai T Miyamoto R Nakagawa E Koichihara R Sakamoto T Mure H. Phenotype variability and allelic heterogeneity in KMT2B-Associated disease. Parkinsonism Relat Disord. (2018) 52:55–61. 10.1016/j.parkreldis.2018.03.022 29653907

[27] Garrido A Simonet C Martí MJ Pérez-Dueñas B Rumià J Valldeoriola F. Good response to bilateral GPI-DBS after 2 years in generalized dystonia due to a mutation in the KMT2B gene (DYT28). Mov Disord. (2018) 33(Suppl. 2). Available online at: https://www.mdsabstracts.org/abstract/good-response-to-bilateral-gpi-dbs-after-2-years-in-generalized-dystonia-due-to-a-mutation-in-the-kmt2b-gene-dyt28/ (accessed April 10, 2023).

[28] Kumar KR Davis RL Tchan MC Wali GM Mahant N Ng K. Whole genome sequencing for the genetic diagnosis of heterogenous dystonia phenotypes. Parkinson Relat Disord. (2019) 69:111–8. 10.1016/j.parkreldis.2019.11.004 31731261

[29] Carecchio M Invernizzi F Gonzàlez-Latapi P Panteghini C Zorzi G Romito L. Frequency and phenotypic spectrum of KMT2B dystonia in childhood: a single-center cohort study. Mov Disord. (2019) 34:1516–27. 10.1002/mds.27771 31216378

[30] Dafsari HS Sprute R Wunderlich G Daimaguler H-S Karaca E Contreras A. Novel mutations in KMT2B offer pathophysiological insights into childhood-onset progressive dystonia. J Hum Genet. (2019) 64:803–13. 10.1038/s10038-019-0625-1 31165786

[31] Cif L Demailly D Lin JP Barwick K Sa M Abela L. KMT2B-related disorders: expansion of the phenotypic spectrum and long-term efficacy of deep brain stimulation. Brain. (2020) 143:3242–61. 10.1093/brain/awaa304 33150406 PMC7719027

[32] Li XY Dai LF Wan XH Guo Y Dai Y Li SL. Clinical phenotypes, genotypes and treatment in Chinese dystonia patients with KMT2B variants. Parkinsonism Relat Disord. (2020) 77:76–82. 10.1016/j.parkreldis.2020.06.002 32634684

[33] Miyata Y Hamanaka K Kumada S Uchino S Yokochi F Taniguchi M. An atypical case of KMT2B-related dystonia manifesting asterixis and effect of deep brain stimulation of the globus pallidus. Neurol Clin Neurosci. (2020) 8:36–8. 10.1111/ncn3.12334

[34] Cao Z Yao H Bao X Wen Y Liu B Wang S. DYT28 responsive to pallidal deep brain stimulation. Mov Disord Clin Pract. (2020) 7:97–9. 10.1002/mdc3.12862 31970221 PMC6962688

[35] Winslow N Maldonado A Zayas-Rodriguez L Lamichhane D. Adult-onset *KMT2B-*related dystonia responsive to deep brain stimulation. Mov Disord Clin Pract. (2020) 7:992–3. 10.1002/mdc3.13093 33163573 PMC7604663

[36] Rajan R Garg K Saini A Kumar M Binukumar BK Scaria V. Pallidal deep brain stimulation for *KMT2B* related dystonia in an indian patient. Ann Indian Acad Neurol. (2021) 24:586–8. 10.4103/aian.AIAN_1316_20 34728955 PMC8513985

[37] Buzo EL De la Casa-Fages B Sánchez MG Sánchez JRP Carballal CF Vidorreta JG. Pallidal deep brain stimulation response in two siblings with atypical adult-onset dystonia related to a KMT2B variant. J Neurol Sci. (2022) 438:120295. 10.1016/j.jns.2022.120295 35635862

[38] Almasy L Bressman SB Raymond D Kramer PL Greene PE Heiman GA. Idiopathic torsion dystonia linked to chromosome 8 in two Mennonite families. Ann Neurol. (1997) 42:670–3. 10.1002/ana.410420421 9382482

[39] Houlden H Schneider SA Paudel R Melchers A Schwingenschuh P Edwards M. THAP1 mutations (DYT6) are an additional cause of early-onset dystonia. Neurology. (2010) 74:846–50. 10.1212/WNL.0b013e3181d5276d 20211909 PMC2839194

[40] Xiao J Zhao Y Bastian RW Perlmutter JS Racette BA Tabbal SD. Novel human pathological mutations. Gene symbol: THAP1 disease: dystonia 6. Hum Genet. (2010) 127:469–70. 21488296

[41] Zech M Boesch S Maier EM Borggraefe I Vill K Laccone F. Haploinsufficiency of KMT2B, encoding the lysine-specific histone methyltransferase 2b, results in early-onset generalized dystonia. Am J Hum Genet. (2016) 99:1377–87. 10.1016/j.ajhg.2016.10.010 27839873 PMC5142117

[42] Bledsoe IO Viser AC San Luciano M. Treatment of dystonia: medications, neurotoxins, neuromodulation, and rehabilitation. Neurotherapeutics. (2020) 17:1622–44. 10.1007/s13311-020-00944-0 33095402 PMC7851280

[43] Kupsch A Benecke R Müller J Trottenberg T Schneider GH Poewe W. Deep-brain stimulation for dystonia study group. Pallidal deep-brain stimulation in primary generalized or segmental dystonia. N Engl J Med. (2006) 355:1978–90. 10.1056/NEJMoa063618 17093249

[44] Parr JR Green AL Joint C Andrew M Gregory RP Scott RB. Deep brain stimulation in childhood: an effective treatment for early onset idiopathic generalised dystonia. Arch Dis Child. (2007) 92:708–11. 10.1136/adc.2006.095380 17460025 PMC2083907

[45] Neychev VK Gross RE Lehéricy S Hess EJ Jinnah HA. The functional neuroanatomy of dystonia. Neurobiol Dis. (2011) 42:185–201. 10.1016/j.nbd.2011.01.026 21303695 PMC3478782

[46] Cury RG Kalia SK Shah BB Jimenez-Shahed J Prashanth LK Moro E. Surgical treatment of dystonia. Expert Rev Neurother. (2018) 18:477–92. 10.1080/14737175.2018.1478288 29781334

[47] Chen W Fan H Lu G. The efficacy and predictors of using GPi-DBS to treat early-onset dystonia: an individual patient analysis. Neural Plast. (2021) 2021:9924639. 10.1155/2021/9924639 34040641 PMC8121596

[48] Udupa K Chen R. The mechanisms of action of deep brain stimulation and ideas for the future development. Progr Neurobiol. (2015) 133:27–49. 10.1016/j.pneurobio.2015.08.001 26296674

[49] Artusi CA Dwivedi A Romagnolo A Bortolani S Marsili L Imbalzano G. Differential response to pallidal deep brain stimulation among monogenic dystonias: systematic review and meta-analysis. J Neurol Neurosurg Psychiatry. (2020) 91:426–33. 10.1136/jnnp-2019-322169 32079672

[50] Haggstrom L Darveniza P Tisch S. Mild parkinsonian features in dystonia: Literature review, mechanisms and clinical perspectives. Parkinson Relat Disord. (2017) 35:1–7. 10.1016/j.parkreldis.2016.10.022 27825543

