# Supplementary material for: GPi DBS treatment outcome in children with monogenic dystonia: a case series and review of the literature
Source: Front Neurol. 2023 Apr 24;14:1151900. doi: 10.3389/fneur.2023.1151900 (PMC10166204; doi:10.3389/fneur.2023.1151900)
Supplement: Supplementary file 4 [file Data_Sheet_1.PDF]

CARE
case report guidelines

CARE Checklist of information to include when writing a case report

(cc) BY-NC-ND

Topic	Item	Checklist item description	Reported on Line
Title	1	The diagnosis or intervention of primary focus followed by the words "case report"	*
Key Words	2	2 to 5 key words that identify diagnoses or interventions in this case report, including "case report"	*
Abstract (no references)	3a	Introduction: What is unique about this case and what does it add to the scientific literature?	*
	3b	Main symptoms and/or important clinical findings	*
	3c	The main diagnoses, therapeutic interventions, and outcomes	*
	3d	Conclusion—What is the main "take-away" lesson(s) from this case?	*
Introduction	4	One or two paragraphs summarizing why this case is unique (**may include** references)	*
Patient Information	5a	De-identified patient specific information	*
	5b	Primary concerns and symptoms of the patient	*
	5c	Medical, family, and psycho-social history including relevant genetic information	*
	5d	Relevant past interventions with outcomes	*
Clinical Findings	6	Describe significant physical examination (PE) and important clinical findings	*
Timeline	7	Historical and current information from this episode of care organized as a timeline	*
Diagnostic Assessment	8a	Diagnostic testing (such as PE, laboratory testing, imaging, surveys).	*
	8b	Diagnostic challenges (such as access to testing, financial, or cultural)	*
	8c	Diagnosis (including other diagnoses considered)	*
	8d	Prognosis (such as staging in oncology) where applicable	*
Therapeutic Intervention	9a	Types of therapeutic intervention (such as pharmacologic, surgical, preventive, self-care)	*
	9b	Administration of therapeutic intervention (such as dosage, strength, duration)	*
	9c	Changes in therapeutic intervention (with rationale)	*
Follow-up and Outcomes	10a	Clinician and patient-assessed outcomes (if available)	*
	10b	Important follow-up diagnostic and other test results	*
	10c	Intervention adherence and tolerability (How was this assessed?)	*
	10d	Adverse and unanticipated events	*
Discussion	11a	A scientific discussion of the strengths AND limitations associated with this case report	*
	11b	Discussion of the relevant medical literature **with references**	*
	11c	The scientific rationale for any conclusions (including assessment of possible causes)	*
	11d	The primary "take-away" lessons of this case report (without references) in a one paragraph conclusion	*
Patient Perspective	12	The patient should share their perspective in one to two paragraphs on the treatment(s) they received	*
Informed Consent	13	Did the patient give informed consent? Please provide if requested	Yes ☐ (circled) No ☐
