# Supplementary material for: GPi DBS treatment outcome in children with monogenic dystonia: a case series and review of the literature
Source: Front Neurol. 2023 Apr 24;14:1151900. doi: 10.3389/fneur.2023.1151900 (PMC10166204; doi:10.3389/fneur.2023.1151900)
Supplement: Supplementary file 5 [file Data_Sheet_2.PDF]

Supplementary Material

GPi DBS treatment outcome in children with Monogenic Dystonia: a case series and review of the literature

Darko Chudy[1,2], Marina Raguž[1,3*], Vladimira Vuletić[4], Valentino Rački[4], Elišа Papić[4], Nataša Nenadić Baranašić[5], Nina Barišić[5]

[1] Department of Neurosurgery, Dubrava University Hospital, Zagreb, Croatia

[2] Department of Surgery, School of Medicine University of Zagreb, Zagreb, Croatia

[3] School of Medicine, Catholic University of Croatia, Zagreb, Croatia

[4] Department of Neurology, School of Medicine, University of Rijeka, Rijeka, Croatia

[5] Department of Pediatrics, University Hospital Centre, Zagreb, Croatia; University of Zagreb, School of Medicine, Zagreb, Croatia

*** Correspondence:**
Marina Raguž
marinaraguz@gmail.com

Supplementary Data - Videos

Video 1. *First part:* Patient I clinical presentation prior to GPi DBS. The patient presented with generalized dystonia at rest and during activity, was not ambulant, with dysphonic and dysarthric speech along with swallowing difficulties. *Second part:* Patient I clinical presentation after GPi DBS. On the first follow up the first patient's signs improved significantly, enabling the patient to sit independently and to walk with the assistance of one person, while in months following DBS, he regained manual abilities, and speech and mobility improvement was registered.

Video 2. *First part:* Patient II clinical presentation prior to GPi DBS. The patient presented with generalized dystonia manifested at rest and provoked by activity. *Second part:* Patient II clinical presentation after GPi DBS. The patient has occasional upper limbs dystonia, and stable gait; he is able to run fast, with voice strength improved, and more fluent and understandable speech.

Video 3. *First part:* Patient III clinical presentation prior to GPi DBS. The patient presented with thoracal kyphosis, occasional jerky arm tremor and coordination disturbances were observed; the patient could not hold extremities in antigravity positions due to muscle weakness. *Second part:* Patient III clinical presentation after GPi DBS. Discrete speech improvement and upper limb function and gait improved so he is able to walk unaided.
