# Supplementary material for: GPi DBS treatment outcome in children with monogenic dystonia: a case series and review of the literature
Source: Front Neurol. 2023 Apr 24;14:1151900. doi: 10.3389/fneur.2023.1151900 (PMC10166204; doi:10.3389/fneur.2023.1151900)
Supplement: Supplementary file 6 [file Data_Sheet_3.PDF]

Supplementary Material

GPi DBS treatment outcome in children with Monogenic Dystonia: a case series and review of the literature

Darko Chudy[1,2], Marina Raguž[1,3*], Vladimira Vuletić[4], Valentino Rački[4], Eliša Papić[4], Nataša Nenadić Baranašić[5], Nina Barišić[5]

[1] Department of Neurosurgery, Dubrava University Hospital, Zagreb, Croatia

[2] Department of Surgery, School of Medicine University of Zagreb, Zagreb, Croatia

[3] School of Medicine, Catholic University of Croatia, Zagreb, Croatia

[4] Department of Neurology, School of Medicine, University of Rijeka, Rijeka, Croatia

[5] Department of Pediatrics, University Hospital Centre, Zagreb, Croatia; University of Zagreb, School of Medicine, Zagreb, Croatia

*** Correspondence:**
Marina Raguž
marinaraguz@gmail.com

Supplementary Table 1. Studies and case reports that reported on patients with DYT-*THAP1* who underwent DBS surgery in the period between 2010 and 2022.

Study	No of patients included	Gender	Age	Anatomical Distribution	Change in BFMDRS-M and -D	DBS target and parameters	Follow up
**Groen et al. 2010**	5 patients;<br><br>4 adult age patients<br>1 pediatric patients	Adult<br>3 ♂ 1 ♀<br>Pediatric<br>1 ♂	Adult<br>6-54 years onset<br>23-70 years surgery<br>Pediatric<br>7 years onset<br>9 years surgery	Severe axial dystonia	Improvement 16-55% overall<br>In pediatric patient<br>M 24>18	Bilateral GPI	unknown
**Jech et al. 2011**	2 patients;<br><br>1 adult age patients<br>1 pediatric patients	Adult<br>1 ♂<br>Pediatric<br>1 ♀	Adult<br>15 years onset<br>20 years surgery<br>Pediatric<br>8 years onset<br>14.5 years surgery	severe generalized dystonia that lapsed into *status dystonicus*	M 41	Bilateral GPI<br>pulse duration 450 ls, frequency 130 Hz, amplitude left 1.6 V, and amplitude right 1.8 V	30 months
**Panov et al. 2012**	1 patients;<br><br>1 pediatric patients	Pediatric<br>1 ♂	Pediatric<br>3 years onset<br>15 years surgery	slurred speech, left side dystonic movements, severe torticollis	M 40>27<br>D 10>6	Bilateral GPI<br>L 3.0V, 150μs, 130Hz<br>R 2.8V, 150μs, 130Hz	8 years
**Krause et al. 2015**	3 patients;<br><br>3 adult age patients<br><br>*members of same family	Adult<br>2 ♂ 2 ♀	Adult<br>7-20 years onset<br>20-41 years surgery	2 patients severe generalized dystonia,<br>1 patient segmental dystonia	Overall >25% improvement	Bilateral GPI	11 years
**Brüggemann et al. 2015**	8 patients;<br><br>8 adult age patients		Adult<br>childhood onset<br>>20 years surgery	5 generalized, 3 segmental dystonia	Overall >40% improvement	GPI, VoA, VIM, STN	Early 1-16months<br>Late 22-92 months

**Vuletic et al. 2016**	1 patients;<br><br>1 pediatric patients	Pediatric<br>1 ♂	Pediatric<br>7 years onset<br>16 years surgery	dystonic movements in the cranio-cervical region (severe latero- and retrocollis, speech and swallowing problems), limbs and trunk anteflexion to 90°	M 52>4.5	Bilateral GPI<br>left amplitude 3.4 V, right amplitude 3.2 V, frequency 130 Hz, and pulse duration 90 µs	2 years
**Danielsson et al 2019**	14 patients;<br><br>7 adult age patients<br>7 pediatric patients	Adult<br>1 ♂ 6 ♀<br>Pediatric<br>4 ♂ 3 ♀	Adult<br>2-8 years onset<br>7-42 years surgery<br>Pediatric<br>4-15 years onset<br>11-17 years surgery	Cervical, leg or foot dystonic movements	M >13-78%	Bilateral GPi, frequency between 90 and 130Hz.	4 yrs 10 months
**Tai et al. 2021**	1 patients;<br><br>1 pediatric patients	Pediatric<br>1 ♂	Pediatric<br>7 years onset<br>14 years surgery	mild hand clumsiness, followed by rapid progression to debilitating scoliosis	M 24>2	Bilateral GPi<br>L 0(−) C(+) 1.0 mA, 450 µs, 130 Hz, R 8(−) C(+) 1.0 mA, 450 µs, 130 Hz	12 months
**Sankhla et al. 2022**	1 patients;<br><br>1 adult age patients	Adult<br>1 ♂	Adult<br>2-8 years onset<br>51 years surgery	dysarthria, involuntary turning of the neck with right torticollis and retrocollis	D 19>4	Bilateral GPi<br>2.9 mA, 210 µs, 130 Hz	12 years
**Grofik et al. 2022**	1 patients;<br><br>1 adult age patients	Adult<br>1 ♂	Adult<br>9 years onset	cervical dystonia with the head rotation; oromandibular dystonia with a dominant impairment of the mimic muscles and tongue; mild jaw opening	M 55><br>30% improvement after six months	Bilateral GPi<br>monopolar setup, 2.9 V, 180 µs, 130 Hz	3 years; postoperative hemorrhage and seizures

Supplementary Table 2. Studies and case reports that reported on patients with DYT-*KMT2B* who underwent DBS surgery in the period between 2017 and 2022.

Study	No of patients included	Gender	Age at onset of symptoms and et surgery	Anatomical Distribution	Change in BFMDRS-M and -D	DBS target and parameters	Follow up
**Meyer et al. 2017**	10 patients;<br>4 adult age patients<br>6 pediatric patients	<u>Adult</u><br>2♂ 2♀<br><u>Pediatric</u><br>4♂ 2♀	<u>Adult</u><br>2-8 years onset<br>19-32 years surgery<br><u>Pediatric</u><br>1-7 years onset<br>7-16 years surgery	mostly dysarthria, dysphonia, swallowing difficulties, torticollis	described improvement for each patient	Bilateral GPI	Over 3 years
**Kawarai et al. 2018**	3 patients;<br>1 adult age patients<br>2 pediatric patients	<u>Adult</u><br>1♀<br><u>Pediatric</u><br>1♂ 1♀	<u>Adult</u><br>8 years onset<br>22 years surgery<br><u>Pediatric</u><br>5 years onset<br>15-16 years surgery	dystonic movements at extremities and trunk, impaired speech	31-73% improvement	Bilateral GPI	Unknown
**Garrido et al. 2018**	1 patient;<br>1 pediatric patient	<u>Pediatric</u><br>1♂	<u>Pediatric</u><br>1 years onset<br>17 years surgery	generalized dystonia, ballistic movements of left arm, bulbar and pyramidal signs	*persistent and excellent response*	Bilateral GPI	2 years
**Kumar et al. 2019**	2 patients;<br>2 adult age patients	<u>Adult</u><br>1♂ 1♀	<u>Adult</u><br>childhood onset<br>20-21 years surgery	generalized dystonia with a tremulous and twisting pattern; severe dysarthria with oromandibular dystonia	*good and moderate response*	Bilateral GPI	Unknown
**Carecchino et al. 2019**	8 patients;<br>1 adult age patients<br>7 pediatric patients	<u>Adult</u><br>1♀<br><u>Pediatric</u><br>1♂ 1♀	<u>Adult</u><br>childhood onset<br>38 years surgery<br><u>Pediatric</u><br>6-11 years onset<br>8-16 years surgery	lower limbs the site of onset of dystonia in 78.5%, progressing to scarcely intelligible speech or anarthria; oromandibular dystonia, generalization	M <38.5%	Bilateral GPI	3 years
**Dafsari et al. 2019**	2 patients;<br>2 pediatric patients	<u>Pediatric</u><br>2♀	<u>Pediatric</u><br>4-6 years onset<br>7-10 years surgery	hyperkinesia of head and trunk; right-handed dystonic tremor and myoclonus	M 28.5>26.5>58<br>M 43>28	Bilateral GPI	11 years<br>11 months
**Zech et al. 2019**	1 patient;<br>1 pediatric patient	<u>Pediatric</u><br>1♀	<u>Pediatric</u><br>childhood onset<br>7 years surgery	generalized dystonia	*restoration from wheelchair-bound state to autonomous ambulation and*	Bilateral GPI	2 years

					marked improvement of fine manual dexterity		
**Cif et al. 2020**	18 patients;<br>3 adult age patients<br>15 pediatric patients	<u>Adult</u><br>3 ♀<br><u>Pediatric</u><br>8 ♂ 7 ♀	<u>Adult</u><br>4-6 onset<br>23-37 years surgery<br><u>Pediatric</u><br>2-9 years onset<br>4.5-18 years surgery	Dysarthria, jaw-opening dystonia, anarthria<br>feeding problems oromandibular dystonia head movements, generalized dystonia	>50% of included subjects presented with M and D improvement<br>>30% at one year follow up	Bilateral GPI	0.25 to 22 years
**Li et al. 2020**	10 patients;<br>3 adult age patients<br>7 pediatric patients	2 ♂ 1 ♀<br><u>Pediatric</u><br>8 ♂ 7 ♀	<u>Adult</u><br>7-12 onset<br>20-36 years surgery<br><u>Pediatric</u><br>2-7 years onset<br>5-17 years surgery	segmental, multifocal or generalized dystonia	M >25-90%	Bilateral GPI<br>1 Bilateral STN	2 to 16 months
**Miyata et al. 2020**	1 patient;<br>1 pediatric patient	<u>Pediatric</u><br>1 ♂	<u>Pediatric</u><br>5 years onset<br>10 years surgery	phasic dystonic movements in the arms and lower limbs, asterixis	*he could easily maintain his arms in the extended position, although asterixis was not completely resolved*	Bilateral GPI	1 year
**Mun et al. 2020**	1 patient;<br>1 adult patient	<u>Adult</u><br>1 ♀	<u>Adult</u><br>27 years onset<br>28 years surgery	right laterocollis and right torticollis with a mobile portion, slowly spread to her trunk and bilateral arms	M 30>9<br>D 11>1	Bilateral GPI<br>L 2.5 mA<br>R 2.6 mA, with 140 µs and 160 Hz	22 months
**Cao et al. 2020**	1 patient;<br>1 pediatric patient	<u>Pediatric</u><br>1 ♂	<u>Pediatric</u><br>7 years onset<br>8 years surgery	generalized dystonia but was able to ambulate dependently	M 73.5>3	Bilateral GPI	7 months
**Winslow et al. 2020**	1 patient;<br>1 adult patient	<u>Adult</u><br>1 ♂	<u>Adult</u><br>childhood onset<br>41 years surgery	hand and head tremor, head rotation and tilt to the right, severe hypertrophy in sternocleidomastoid and trapezii muscles, but no dysphonia or abnormal facial features	M 35>4<br>D 4>1	Bilateral GPI<br>2.5 mA, 120 µs, 130 Hertz<br>(R E10-11-12, L E2-3-4)	1 month

**Abel et al. 2021**	1 patient;<br>1 pediatric patient	Pediatric<br>1 ♂	Pediatric<br>3 years onset<br>8 years surgery	generalized dystonia and severe dysarthria	M 59>9<br>D 22>1	Bilateral GPI<br>2 mA on both sides with a pulse width of 80 µs and a frequency of 130 Hz	16 month
**Rajan et al. 2021**	1 patient;<br>1 pediatric patient	Pediatric<br>1 ♀	Pediatric<br>11 years onset<br>17 years surgery	generalized dystonia involving the lower face, tongue, neck, trunk, and all four limbs; anarthric and wheelchair bound	M 94>57.5<br>D 24>22	Bilateral GPI<br>L 3(-) C (+), 1.5 V/60 µs/150 Hz<br>R11(-) C (+), 1.7V/90 µs/150 Hz	6 months
**Buzo et al. 2022**	2 patients	Adult	Adult onset	cervical dystonia			
